# Realization of Multifunctional Metamaterial Structure Based on the Combination of Vanadium Dioxide and Graphene

**DOI:** 10.3390/nano12162883

**Published:** 2022-08-22

**Authors:** Mingxuan Cao, Junchao Wang, Matthew M. F. Yuen, Dexian Yan

**Affiliations:** 1 Department of Intelligent Manufacturing, Wuyi University, Jiangmen 529020, China; 2 Department of Mechanical Engineering, Hong Kong University of Science and Technology, Hong Kong 999077, China; 3 Key Laboratory of Electromagnetic Wave Information Technology and Metrology of Zhejiang Province, College of Information Engineering, China Jiliang University, Hangzhou 310018, China

**Keywords:** metamaterial, functional material, polarization conversion, absorber, filter

## Abstract

Combining tunable properties and various functionalities into a single metamaterial structure has become a novel research hotspot and can be used to tackle great challenges. The multifunctional metamaterial structure that combines absorption, linear-to-circular (LTC) polarization conversion, filtering and switching functions into a single metamaterial device was designed and investigated in this study. The switching of different functions can be achieved based on the phase transition of vanadium dioxide (VO_2_) and change of graphene chemical potential. When VO_2_ is in a metal state, the multi-frequency absorption and LTC polarization conversion can be achieved with different chemical potentials. When VO_2_ is in the insulator state and the polarization angle of incident wave is 45°, the device can be used to select or isolate the incident waves with different polarization states in the frequency region of 1.2–1.8 THz. Furthermore, when the chemical potentials are 0.05 eV and 1.2 eV, the corresponding transmissions of the TE-polarized wave demonstrate the opposite results, realizing the switching functions in the frequency region of 0.88–1.34 THz. In the frequency region above 2 THz, the multi-frequency rejection filter can be achieved. The designed switchable multifunctional metamaterial device can be widely implemented in radar monitoring and communication systems.

## 1. Introduction

Modulating the terahertz waves is particularly important in responding to the increasingly complex electromagnetic environment and communication needs. The absorber, polarization converter, and filter are especially essential devices in the fields of wave modulation. Metamaterials are artificially designed electromagnetic materials that are formed by periodic distribution of subwavelength microstructures. Traditional metamaterial devices, including absorbers, polarization converters, filters, etc., are usually designed for realizing a single function. In recent years, with the introduction of functional materials such as graphene, VO_2_, liquid crystal, etc., the ability to design multifunctional metamaterial structures has become one of the main research hotspots [1,2,3,4,5,6,7,8,9,10]. Among these materials, graphene can provide the excellent electrical properties, including strict field confinement, high electron mobility, flexibility, and tuning properties [11]. Graphene-based metamaterial devices enable efficient dynamic modulation, since the graphene conductivity can be dynamically modulated by changing the chemical potential through electrical stimulating or chemical doping [12]. One method to realize function reconfiguration is to introduce the graphene materials into the metamaterial structures. In addition, VO_2_ is a kind of phase-change material that performs the reversible transition from the insulating phase to the metal phase excited by the electric field, temperature field, and light field. Thus, VO_2_ enables the switchable and tunable functionalities for smart devices since the conductivity is capable of 4–5 orders of magnitude change, and the phase-change process can be realized in several picoseconds [13]. Inspired by the above extraordinary properties, VO_2_ can be used as a promising candidate in several advanced devices, such as optical switches, modulators, absorbers, and polarization converters.

Advances toward the combination of VO_2_ and graphene in multifunctional metamaterial structures have been put forward. By combing the VO_2_ and graphene, Zhu et al. designed a switchable terahertz metamaterial structure to realize wide-band and multi-band absorption [14]. Liu et al. demonstrated a metamaterial absorber by treating the VO_2_ and graphene to realize dynamically tunable dual-wide-band absorption [11]. When the VO_2_ switches from the metallic phase to the insulator phase, the operation frequency can be changed from the high-frequency to low-frequency region accordingly. By altering the Fermi energy level of graphene, its absorptivity can be continuously varied from 5.2% to 99.8%. Zhao et al. proposed a dynamically switched wide-band metamaterial polarization converter by treating the VO_2_ and graphene gratings in terahertz region [15]. The phase transition of VO_2_ enables the polarization conversion switching between transmissive and reflective modes, and the introduction of double-layer graphene grating should improve the transmittance. Tang et al. designed a multifunctional terahertz metamaterial structure based on the hybrid VO_2_-graphene integrated structure [16]. Enabled by the phase transition of VO_2_, the asymmetric transmission and two different polarization conversions can be integrated into one device in the terahertz region. Wang et al. realized a dual-functional tunable wide-band absorber/reflector based on the periodic cross-shaped graphene array and a VO_2_ layer [17]. Most of the reported studies have focused on achieving similar functionality in a single device by using a combination of VO_2_ and graphene.

Within the investigation of this study, we aimed to design a multifunctional metamaterial structure based on the manipulability of the chemical potential of graphene and the phase-transition properties of VO_2_ material. Compared to the previously reported metamaterial devices, the proposed scheme here can provide three main advantages, which are as follows: (1) different functions, including multi-frequency absorption and linear-to-circular (LTC) polarization conversion, and different transmission characteristics can be integrated into a single metamaterial structure; (2) when VO_2_ is in the metal state, the designed structure works in reflection mode, and the functions of the metamaterial structure can switch between absorption (*µ*_c_ = 1.2 eV) and LTC polarization conversion (*µ*_c_ = 0.05 eV) by varying the graphene chemical potential; (3) when VO_2_ is in the insulator state, the structure works in the transmissive mode, which exhibits different transmission characteristics for different incidences. In addition, the effects of several structure geometrical parameters on the performances have also been investigated. The proposed scheme may pay a new route in designing tunable and switchable multifunctional terahertz metamaterial structures.

## 2. Materials and Methods

**Figure 1:**
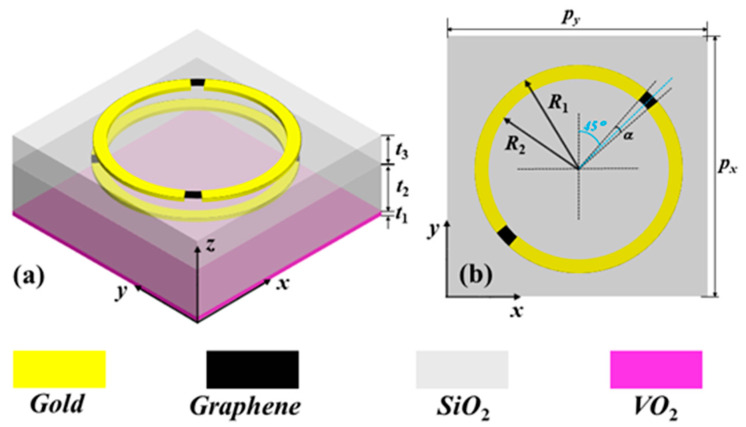
Proposed metamaterial structure: (**a**) three-dimensional schematic and (**b**) top view.

Figure 1 gives the structural diagram of the proposed hybrid VO_2_–graphene-integrated metamaterial structure, which is arranged periodically in the *x*- and *y*-direction. The unit cell of the proposed metamaterial structure is composed of five layers from top to bottom: metal split ring resonator, SiO_2_ spacer, metal split ring resonator, SO_2_ spacer, and VO_2_ layer. As depicted in Figure 1, the structure sizes of the unit cell are *p*_x_ = *p*_y_ = 80 µm, *t*_1_ = 0.2 µm, *t*_2_ = 24 µm, *t*_3_ = 13 µm, *R*_1_ = 30 µm, *R*_2_ = 27 µm, and *α* = 5°. The top and middle metal split rings are 0.2 µm–thick gold microstructures with a conductivity of 4.561 × 10^7^ S/m [18]. The gaps of the two metal split rings are arranged vertically, and the gaps are filled with graphene material with a thickness of 1 nm [18]. In the investigation, the dielectric layers are SiO_2_ material with a dielectric constant of *ε*_r_ = 3.75 [15] and electric tand. of 0.0004. The conductivity of graphene and VO_2_ can be varied by using voltage and temperature, respectively.

The fast-developing micro–nano-fabrication technology can be used to fabricate the designed multifunctional structures [15]. The designed metamaterial device can be manufactured by using the techniques in the related references [19,20,21].

In the terahertz frequency region, the surface complex conductivity of graphene, *σ*_g_ = *σ*_intra_ + *σ*_inter_, can be characterized by the intra-band conductivity, *σ*_intra_, and the inter-band conductivity, *σ*_inter_ [22,23]:

(1) $$\sigma_{\mathrm{intra}}=-j\frac{e^{2}k_{B}T}{\pi \hbar^{2}(\omega -j2\Gamma )}\left(\frac{\mu_{c}}{k_{B}T}+2\ln\left(e^{-\frac{\mu_{c}}{k_{B}T}}+1\right)\right)$$

(2) $$\sigma_{\mathrm{inter}}=-j\frac{e^{2}}{4\pi \hbar }\ln\left(\frac{2|\mu_{c}|-(\omega -j2\Gamma )\hbar }{2|\mu_{c}|+(\omega -j2\Gamma )\hbar }\right)$$

where *ω* represents the angular frequency of the incident terahertz wave, *µ*_c_ represents the chemical potential or Fermi level of graphene [15], *T* denotes the temperature, *e* represents the electronic charge, *k*_B_ denotes the Boltzmann constant, Γ = *ħ*/2π denotes the phenomenology scattering rate, *ħ* represents the reduced Plank constant, *τ* = *µµ*_c_/(e$v_{f}^{2}$) represents the relaxation time, and here *τ* = 0.2 ps is used in our study [24,25]. Moreover, *v*_f_ = 1.1 × 10^6^ m/s represents Fermi velocity, and *μ* represents the carrier mobility, which is mainly influenced by the graphene doping degree or its own defects at room temperature [26]. The surface conductivity of monolayer graphene is affected by the chemical potential variations caused by the chemical doping [27] or bias voltage stimulating [28]. Based on the Pauli exclusion principle, the *σ*_inter_ can be ignored when *ħω* << *µ*_c_ and *k*_B_*T* << *µ*_c_ [29]. Thus, the surface conductivity, *σ*_g_, of graphene can be simply expressed as the Drude model [23]:

(3) $$\sigma_{g}\approx \frac{e^{2}\mu_{c}}{\pi \hbar^{2}}\frac{j}{\omega +j/\tau }$$

The effective permittivity, *ε_g_*, of the graphene can be described by *ε_g_* = 1 + *jσ*_g_/(*ε*_0_*ωd*_g_) [18,30], where *ε*_0_ represents the vacuum permittivity, and *d*_g_ denotes the thickness of the graphene material, which is set as 1 nm in the investigation [18].

Besides graphene, VO_2_ is one of the important phase-transition materials. In general, the permittivity of VO_2_ in the terahertz region can be characterized by using the Drude model [31]:

(4) $$\varepsilon_{VO_{2}}=\varepsilon_{\infty }-\frac{\omega_{p}^{2}(\sigma_{VO_{2}})}{\omega^{2}+i\gamma \omega }$$

where *ε*_∞_ = 12 represents the permittivity at infinite frequency; *γ* = 5.75 × 10^13^ rad/s. *ω_p_*($\sigma_{{\mathrm{VO}}_{2}}$) represents the plasma frequency; and $\omega_{p}^{2}$($\sigma_{{\mathrm{VO}}_{2}}$) = $\sigma_{{\mathrm{VO}}_{2}}$/($\sigma_{{\mathrm{VO}}_{2}}$$\omega_{p}^{2}$(*σ*_0_)), where *ω_p_*(*σ*_0_) = 1.4 × 10^15^ rad/s. To simulate the insulating-metal phase change characteristics of VO_2_, different conductivities are used to characterize the different optoelectronic characteristics of VO_2_. In this study, $\sigma_{{\mathrm{VO}}_{2}}$ = 3 × 10^5^ S/m and $\sigma_{{\mathrm{VO}}_{2}}$ = 10 S/m [32] were used to represent the metallic and insulator states of VO_2_, respectively. The conductivity of VO_2_ can be changed by laser radiation, external bias voltage, and thermal control.

The metamaterial device was designed and the related simulations were performed by using the finite difference time-domain method. The unit cell boundary conditions are used in *x* and *y* axes to mimic the infinite arrays, and the open boundaries are applied in the *z* axis. Linearly and circularly polarized terahertz waves transmitting along the -*z* direction are incident on the metamaterial structure.

## 3. Results and Discussions

The operation characteristics of the designed multifunctional metamaterial unit cell structure were studied, and the electromagnetic responses of different functions were achieved. By combining the phase transition of VO_2_ and variation of graphene chemical potential, different functions can be integrated into a single metamaterial structure. When VO_2_ is in the metallic state ($\sigma_{{\mathrm{VO}}_{2}}$ = 3 × 10^5^ S/m), according to different chemical potentials, the designed structure can exhibit linear-to-circular (LTC) polarization conversion properties (*µ*_c_ = 0.05 eV) and absorption properties (*µ*_c_ = 1.2 eV), respectively. In 2017, Bezares et al. presented FeCl_3_-intrcalated graphene as a new plasmonic material with high stability under environmental conditions and carrier concentrations, corresponding to a Fermi energy of 1.21 eV on the two-monolayer sample and 1.4 eV for the bilayer graphene [33]. By use of the electrolyte gate technique, for example, carrier densities as high as *n* = 4 × 10^14^ cm^−2^ have been obtained for electrons and holes [34], and the corresponding chemical potential is about 1.17 eV [35]. A reasonable tuning range of the chemical potential can reach 1.2 eV based on the current situation that a carrier density of up to 10^14^ cm^−2^ has been realized in experiments [36,37]. When VO_2_ is in the insulator state ($\sigma_{{\mathrm{VO}}_{2}}$ = 10 S/m), the designed metamaterial structure can act as a filter, as well as a switch.

### 3.1. Designed Metamaterial Structure Works as the Polarization Converter

**Figure 2:**
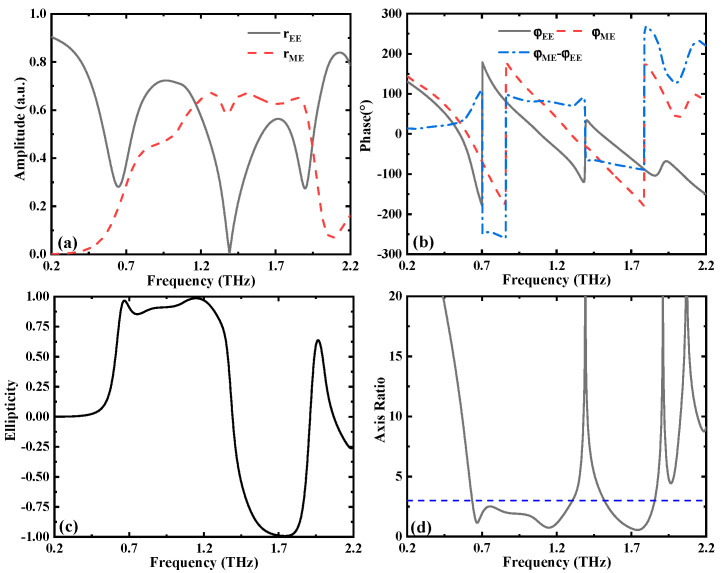
Calculated (**a**) reflection coefficients amplitudes and (**b**) phases (phase difference) for TE-polarized normal incident terahertz wave. Calculated (**c**) ellipticity and (**d**) axis ratio of the structure with TE polarization incident.

When the conductivity of VO_2_ layer is $\sigma_{{\mathrm{VO}}_{2}}$ = 3 × 10^5^ S/m, the VO_2_ layer acts like a metal that prevents the transmission of incident terahertz waves. In this case, when the chemical potential of graphene is set to *µ*_c_ = 0.05 eV, the designed metamaterial structure can be used as an LTC polarization converter, which is illuminated by the TE-polarized (electric field polarized along the *y* axis) or TM-polarized (electric field polarized along the *x* axis) incident terahertz waves with a polarization angle of 0°. In the *x–y*-coordinate system, for realizing the LTC polarization conversion, the reflection coefficients for co-polarization (TE-to-TE), *r_EE_*, and cross-polarization (TE-to-TM), *r_ME_*, should be equal, providing a phase difference of Δ*φ* = *φ_ME_* − *φ_EE_* = 2*n*π ± π/2 (*n* represents an integer), and “−” and “+” define the left-handed and right-handed circularly polarized terahertz waves [38,39]. As given in Figure 2a,b, in the frequency bands of 0.64–1.28 THz and 1.54–1.85 THz, the reflection coefficients exhibit approximately the same intensity, and the corresponding phase difference is around 90° (or −270°) and −90°. Thus, the reflective wave can be regarded as the right-handed circularly polarized (RHCP) wave and left-handed circularly polarized (LHCP) wave in the corresponding frequency region. To quantify the performance of the designed metamaterial structure, Stokes parameters (S) are used to evaluate the efficiency of the LTC polarization conversion. The expressions of the Stokes parameters are as follows [40]:

(5) $$S_{0}=|r_{EE}{|}^{2}+|r_{ME}{|}^{2}$$

(6) $$S_{1}=|r_{EE}{|}^{2}-|r_{ME}{|}^{2}$$

(7) $$S_{2}=2|r_{EE}||r_{ME}|\cos(\Delta \varphi )$$

(8) $$S_{3}=2|r_{EE}||r_{ME}|\sin(\Delta \varphi )$$

The normalized ellipticity of *χ* = *S*_3_/*S*_0_ is further given to evaluate the polarization conversion degree. Specifically, *χ* = −1 and *χ* = +1 indicate that the reflective wave is a LHCP wave and RHCP wave, respectively. Figure 2c depicts the relationship between the normalized ellipticity and the operating frequency. The results demonstrate that the ellipticity approaches 1 within the frequency range of 0.64 to 1.28 THz (ellipticity above 0.85), which further confirms that the reflective wave can be estimated as RHCP wave. In addition, in the frequency range of 1.54 to 1.85 THz, the ellipticity approaches −1 (below −0.85), indicating that the reflective wave can be regarded as the LHCP wave.

In addition, we define sin(2*β*) = *S*_3_/*S*_0_ and introduce the axis ratio AR = 10log(tan*β*) to evaluate the circular polarization characteristics [41], where *β* represents the ellipticity angle. As shown in Figure 2d, the AR value is lower than 3 dB in the frequency bands of 0.64 to 1.28 THz and 1.54 to 1.85 THz, demonstrating that the proposed structure realizes good LTC polarization conversion operation properties. The energy conversion efficiency is calculated by using the equation *η* = |*r_ME_*|^2^ + |*r_EE_*|^2^. The efficiency of the RHCP is higher than 0.8, and that of the LHCP is larger than 0.7. The results demonstrate that the designed metamaterial structure can realize the high-efficiency conversion operation.

**Figure 3:**
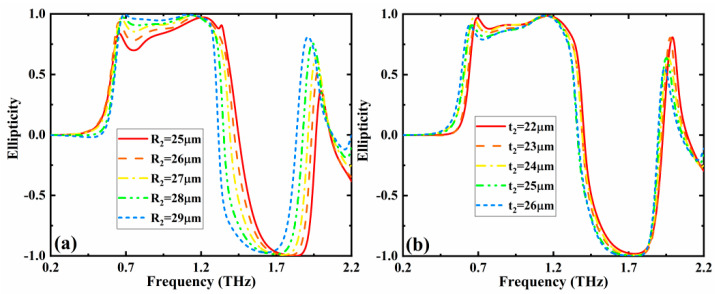
Effects of different (**a**) *R*_2_ and (**b**) *t*_2_ on the ellipticity when the conductivity of VO_2_ and chemical potential of graphene are $\sigma_{{\mathrm{VO}}_{2}}$ = 3 × 10^5^ S/m and *µ*_c_ = 0.05 eV, respectively.

In general, the geometry parameters of the metamaterial device have an effect on the operation performances. In addition, errors can be introduced in the structural dimensional parameters during the device processing. Therefore, the geometric parameters of the metamaterial structure were also investigated. The variations of the ellipticity spectrum of the LTC polarization conversion structure with the geometry parameters (*R*_2_ and *t*_2_) were studied when the other parameters are fixed as initial settings, and the results are given in Figure 3. From Figure 3a, we can see that, when the value of *R*_2_ rises from 25 to 29 µm, the ellipticity of RHCP wave increases. At the same time, the corresponding bandwidth narrows slightly, which is caused by the red-shift of the overall LHCP frequency band with the augment of *R*_2_. Furthermore, as given in Figure 3b, the overall ellipticity spectrum exhibits a slight red-shift trend with the augment of *t*_2_. The above results demonstrate that the variation of geometric parameters have a certain influence on the operating characteristics, but the influence is acceptable according to the fabricating error.

### 3.2. Designed Metamaterial Structure Works as the Absorbers

**Figure 4:**
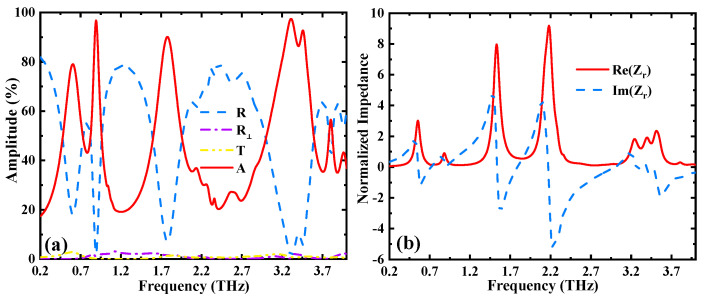
(**a**) Reflectance spectrum, transmittance spectrum, and absorption spectrum of the metamaterial structure; (**b**) real and imaginary part of the relative impedance, with $\sigma_{{\mathrm{VO}}_{2}}$ = 3 × 10^5^ S/m and *µ*_c_ = 1.2 eV.

Using the above metamaterial structure operating at reflection mode ($\sigma_{{\mathrm{VO}}_{2}}$ = 3 × 105 S/m), when the chemical potential of graphene is assumed to be 1.2 eV, the metamaterial device is able to obtain selective absorption of linearly and circularly polarized incident terahertz waves. Thus, the metamaterial device can achieve the multi-frequency absorption. Firstly, we demonstrate the absorption operation of the metamaterial structure under linear polarization incident. It should be noted that the results exhibit the same response for the TM-polarized terahertz waves incidence because of the symmetry of the structure. The absorption, *A*, of the designed metamaterial structure can be calculated from the expression *A* = 1 − *R* − *T* − *R*_⊥_. In the above expression, *R* = |*S*_11_|^2^ and *T* = |*S*_21_|^2^ represent the reflection and transmission, respectively. Moreover, the parameter *R*_⊥_, indicating the reflection of cross-polarized wave, also needs to be taken into account [42]. In the investigation, on the basis of the calculation results illustrated in Figure 4a, the transmission *T* and cross-polarized reflection *R*_⊥_ can be neglected. The TE-polarized incident terahertz wave exhibits three absorption peaks at 0.89 THz, 1.78 THz, and 3.31 THz, and the corresponding absorptivity values of the three peaks are 96.77%, 90.14% and 97.44%, respectively. When the incident electric field is along the *y* direction, the strong electric polarization can be first caused, forming an electric dipole resonance. Then the incident waves enter the SiO_2_ layer, and the ground metallic phase VO_2_ film induces destructive interference that resembles the Fabry–Perot resonant effect, leading to a huge energy dissipation of the incoming terahertz wave.

When the terahertz wave is vertically incident on the metamaterial structure’s surface, the intrinsic mechanism for the operation of metamaterial absorber can be explained by using the impedance matching theory. The real and imaginary parts of the relative impedance of the metamaterial absorber can be derived based on the S parameters. The expressions of the relative impedance are as follows [9]:

(9) $$A=1-R=1-{\left|\frac{Z-Z_{0}}{Z+Z_{0}}\right|}^{2}=1-{\left|\frac{Z_{r}-1}{Z_{r}+1}\right|}^{2}$$

(10) $$Z_{r}=\pm \sqrt{\frac{{\left(1+S_{11}\right)}^{2}-S_{21}^{2}}{{\left(1-S_{11}\right)}^{2}-S_{21}^{2}}}$$

where *Z*_0_ and *Z* represent the effective impedances in air and in the metamaterial device, respectively. Moreover, *Z_r_* = *Z/Z*_0_ represents the relative impedance between the air and the metamaterial device. The absorption of the metamaterial device reaches the maximum values when the impedance of the device is equal to the impedance in the air, meaning that the relative impedance, *Z_r_*, of the structure is equal to 1. As demonstrated in Figure 4b, the real part is gradually approaching 1, while the imaginary part is gradually approaching 0 at the corresponding high absorption resonant peaks, indicating that the effective impedance of the structure and the air gradually match each other. It should be noted that, when the LHCP or RHCP terahertz waves are incident on the device, the absorption performances of the device are similar to the linearly polarized incidence (not shown in the paper).

**Figure 5:**
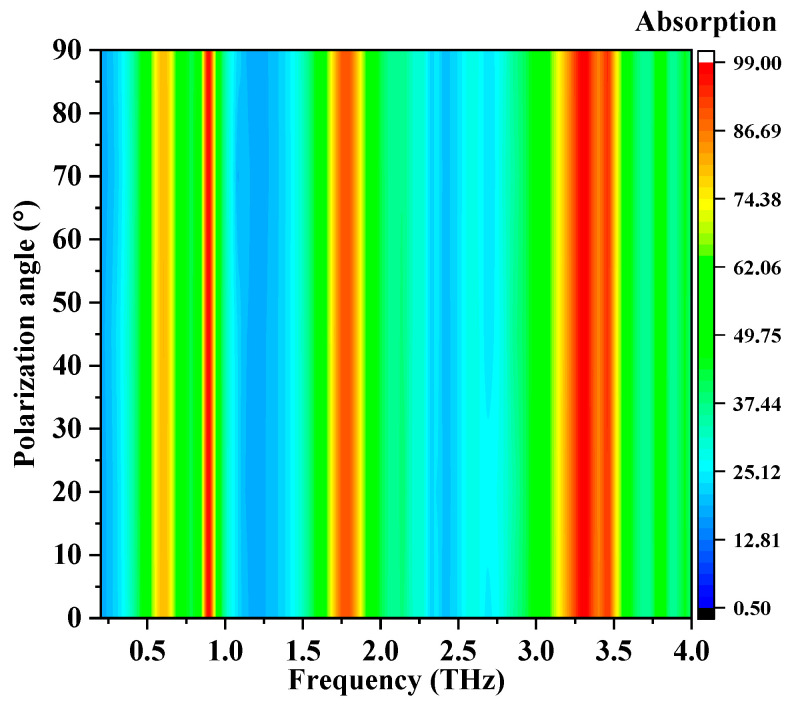
Absorption versus frequency with different polarization angles.

Next, the absorption properties of the multi-band metamaterial absorber with different polarization angles are studied. Figure 5 depicts the absorption versus frequency as the polarization angle rises from 0° to 90° in a step of 5°. The results demonstrate that the operation responses of the proposed multi-band metamaterial absorber with TE-polarized normal incidence do not change with the variation of the polarization angle, and the absorption spectra are highly coincident. The insensitivity to the polarization angle is caused by the symmetry structure of the unit cell in the *x* and *y* axes, and it is very helpful in many practical applications.

**Figure 6:**
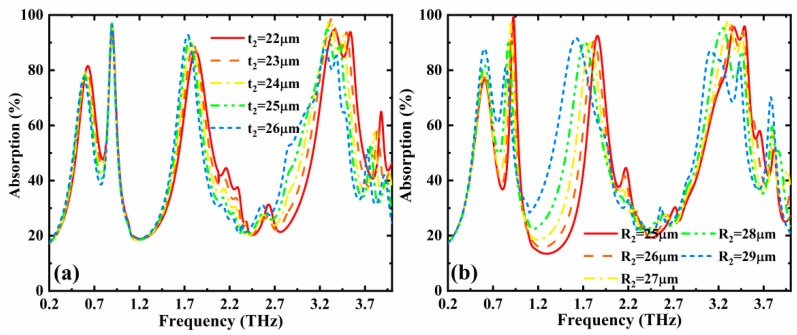
Absorption versus operation frequency with different (**a**) *R*_2_ and (**b**) *t*_2_ when the conductivity of VO_2_ and chemical potential of graphene are $\sigma_{{\mathrm{VO}}_{2}}$ = 3 × 10^5^ S/m and *µ*_c_ = 1.2 eV, respectively.

In addition, we also discussed the effects of the geometric parameters of *t*_2_ and *R*_2_ on the absorption performances. As illustrated in Figure 6a, with the increase of *t*_2_, the frequency bands around 1.78 THz and 3.35 THz show a slight red-shift trend. From Figure 6b, we can see that, when *R*_2_ rises from 25 to 29 µm, the frequency band around 1.7 THz illustrates a more obvious change in the absorption performance than that of the other frequency bands. Moreover, the overall absorption spectrum demonstrates a red-shift trend. 

### 3.3. Designed Metamaterial Structure Acts as a Filter

**Figure 7:**
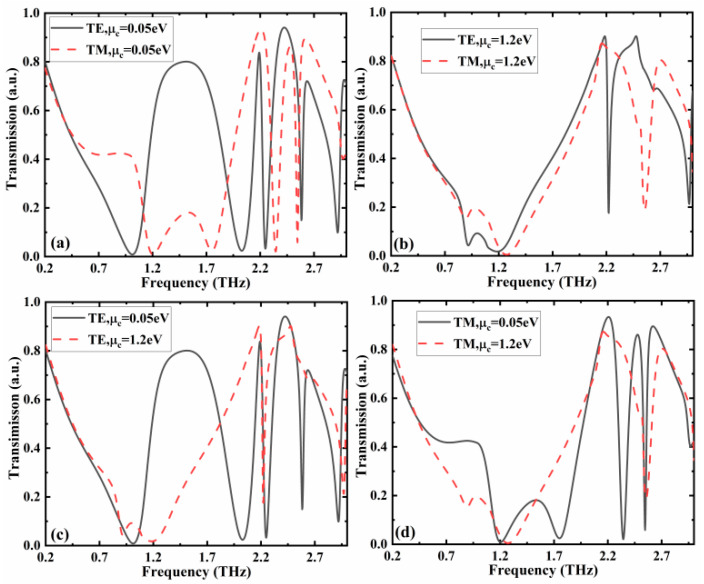
Transmission characteristics of the designed metamaterial structure when the conductivity of VO_2_ is 10 S/m and polarization angle of incident wave is 45°. Transmissions of TE- and TM-polarized incident wave when *µ*_c_ is (**a**) 0.05 eV and (**b**) 1.2 eV. Transmissions of (**c**) TE- and (**d**) TM-polarized incident wave when *µ*_c_ are 0.05 and 1.2 eV.

When the conductivity of VO_2_ is 10 S/m and the chemical potentials of graphene are 0.05 and 1.2 eV, the designed metamaterial structure is able to act as a filter, which performs different filtering responses for TE- and TM-polarized incident terahertz waves with the polarization angle of 45°. The device contains a top layer pattern combining metal split ring and graphene filled in gaps, a first lossy SiO_2_ dielectric layer, a middle layer pattern with the same structure as the top layer, a second lossy SiO_2_ dielectric layer, and a base VO_2_ dielectric layer. For different graphene chemical potential values, the responses related to the TE- and TM-polarized terahertz waves are different. When the chemical potential is *µ*_c_ = 0.05 eV, the TE-polarized terahertz waves and TM-polarized terahertz waves have completely different transmission rates in the low frequency band, as depicted in Figure 7a. In the frequency band of 1.2–1.8 THz, the maximum transmission of the TE-polarized incident wave is higher than 80%, while the value of the TM-polarized incident wave is lower than 20%. This phenomenon can be used to distinguish the incident terahertz waves with different polarization states and can also act as an isolator for incident waves with different polarization states. In the frequency region higher than 2 THz, the high transmission of 83.8% and 94% can be achieved at the center frequencies of 2.19 and 2.42 THz for the TE-polarized incident wave, and the high transmissions of 93.4%, 86%, and 89% can be achieved at the center frequencies of 2.2, 2.47, and 2.62 THz for the TM-polarized incident wave, respectively. In addition, at the resonance frequencies, *f*_c_, of 2.25 THz (TE-polarization), 2.34 THz (TM-polarization), and 2.54 THz (TM-polarization), the corresponding transmissions of the proposed metamaterial structure are 3.2%, 2.1%, and 5.8%, demonstrating good stop-band characteristics. The corresponding stop-band bandwidths ∆*f*(−3 dB) are 120, 140, and 50 GHz, and the corresponding quality factors, *Q* = *f*_c_/∆*f*, are 18.75, 16.71, and 50.8. Moreover, the transmission properties can also enable the construction of a terahertz wave sensor, and the related investigation method can be found in our previous work [43].

When the chemical potential is *µ*_c_ = 1.2 eV, the TE-polarized terahertz waves and TM-polarized terahertz waves exhibit similar transmission responses in the low frequency region, as given in Figure 7b. In the high frequency range, the TE- and TM-polarized incident waves have a notch resonant frequency point, with the transmission values of 17% (2.22 THz) and 18% (2.56 THz), respectively. For TE-polarized incident wave, the designed structure can provide switching functions in a specific frequency range, as illustrated in Figure 7c. In the low frequency region, when the chemical potentials are 0.05 and 1.2 eV, the corresponding transmissions of the TE-polarized wave demonstrate the opposite results, leading to the realization of switching function. When *µ*_c_ is 1.2 eV, the transmittance of TE-polarized incident wave is lower than 10% in a narrow frequency range (0.88–1.34 THz). It should be noted that the bandwidth can be broadened by further optimization, including changing the structure size and material properties, etc. In practical applications, specific functions can be implemented according to the requirements. In the high frequency range, when *µ*_c_ augments from 0.05 to 1.2 eV, the metamaterial structure changes from a multi-frequency rejection filter to a single-frequency rejection filter. As given in Figure 7d, for TM-polarized incident wave, when *µ*_c_ rises from 0.05 to 1.2 eV, the structure also changes from a multi-frequency rejection filter to a single-frequency rejection filter.

**Figure 8:**
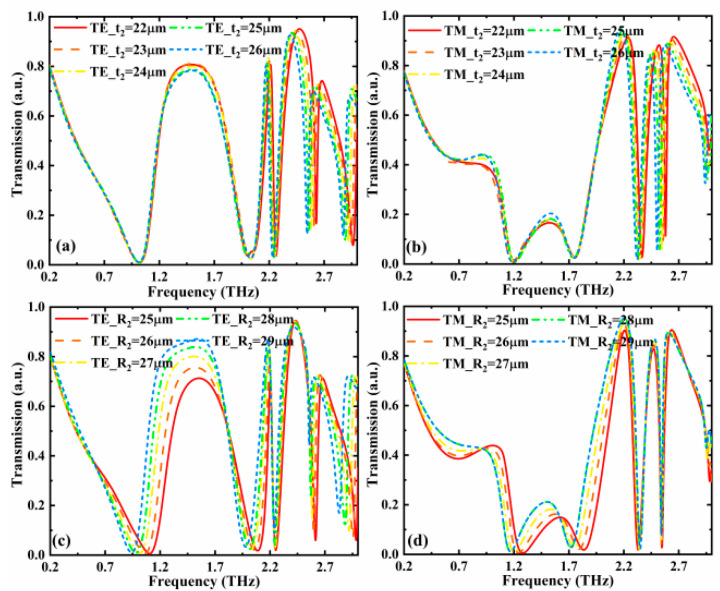
Transmission versus operation frequency with different *t*_2_ for (**a**) TE- and (**b**) TM-polarized incident terahertz wave and different *R*_2_ for (**c**) TE- and (**d**) TM-polarized incident terahertz wave when the conductivity of VO_2_ and chemical potential of graphene are $\sigma_{{\mathrm{VO}}_{2}}$ = 10 S/m and *µ*_c_ = 0.05 eV and the polarization angle of incident wave is 45°.

In addition, the influences of geometric parameters (*t*_2_ and *R*_2_) on the filtering characteristics were also investigated. The conductivity of VO_2_ and chemical potential of graphene are $\sigma_{{\mathrm{VO}}_{2}}$ = 10 S/m and *µ*_c_ = 0.05 eV, respectively, and the polarization angle of incident wave is 45°. As given in Figure 8a,b, in the low frequency region (below 2 THz), the changes of *t*_2_ result in only a slight effect on the transmission intensity. In the high frequency band, the change of *t*_2_ can affect the position of the resonant peaks, causing the transmission spectrum to show a red-shift trend with the augment of *t*_2_. As illustrated in Figure 8c, for the TE-polarized incident wave, when the *R*_2_ rises from 25 to 29 µm, the transmission of the frequency band 1.2–1.8 THz gradually increases, and the frequency band has a red-shift trend. In Figure 8d, the transmission of the TM-polarized incident wave in the frequency band 1.2–1.8 THz slightly increases with the increase of *R*_2_, and the position of the notch resonant frequency does not change significantly. The results demonstrate that the above transmission functions can also be realized when the geometric parameters perform a slight variation.

## 4. Conclusions

In conclusion, we proposed a novel kind of multifunctional metamaterial structure based on the combination of VO_2_ and graphene materials that performs the absorption, LTC polarization conversion, filtering, and switch functions in a single metamaterial device. The integration of different functions into a single metamaterial device was realized by using the insulator–metal phase transition of VO_2_ and variation of graphene chemical potential. When VO_2_ is in metal state and the chemical potential is 0.05 eV, the RHCP and LHCP can be achieved in the frequency bands of 0.64–1.28 THz and 1.54–1.85 THz, respectively. When the chemical potential rises from 0.05 to 1.2 eV, three absorption peaks at 0.89, 1.78, and 3.31 THz with the corresponding absorptivity of 96.77%, 90.14%, and 97.44% can be obtained. When VO_2_ is in insulator state and the polarization angle of incident wave is 45°, the maximum transmission of the TE-polarized wave and TM-polarized wave are different in the range of 1.2–1.8 THz, which can be used to distinguish different polarized incident waves. The corresponding transmissions of the TE-polarized wave demonstrate the opposite results with the different chemical potential of graphene, realizing the switching functions in the frequency band of 0.88–1.34 THz. When the operation frequency is above 2 THz, the multi-frequency rejection filter can be achieved. The proposed metamaterial structure can be applied in many advanced fields, including the fronthaul of terahertz communication system, electromagnetic stealth, radar, etc.

## Data Availability

The data presented in this study are available upon request from the corresponding author.
